# Dexamethasone, Dexmedetomidine, and Combination of Dexamethasone–Dexmedetomidine as Adjuvants to Bupivacaine for Costoclavicular Block: A Randomized Controlled Study

**DOI:** 10.1155/anrp/5683873

**Published:** 2025-06-27

**Authors:** Keerthana Kalaimani, Anisha Pauline Paul, Aruna Parameswari, Mahesh Vakamudi, Varun Karuppiah Thiagarajan, Kishore Manivannan

**Affiliations:** Department of Anesthesiology, Sri Ramachandra Medical College and Research Institute, Sri Ramachandra Institute of Higher Education and Research (SRIHER), Porur, Chennai, Tamil Nadu, India

**Keywords:** costoclavicular block, dexamethasone, dexmedetomidine

## Abstract

**Background:** The costoclavicular block is an upcoming approach in blocking the brachial plexus for upper limb surgeries. The addition of dexamethasone and dexmedetomidine to the local anesthetic mixture can prolong the duration of analgesia of brachial plexus block. We compared the addition of three different adjuvants—dexamethasone, dexmedetomidine, and dexamethasone—dexmedetomidine combination with bupivacaine in costoclavicular block.

**Methods:** We randomized 105 patients undergoing elective hand and forearm surgery under ultrasound guided costoclavicular block. Along with the local anesthetics, Group D patients received 4 mg dexamethasone, Group X patients received 1 µg/kg dexmedetomidine, and Group D-X patients received 4 mg dexamethasone and 1 µg/kg dexmedetomidine. The primary outcome analyzed was the analgesic duration. The secondary outcomes studied were the duration of sensory and motor block, time to onset of sensory and motor block, sedation scores, and adverse effects.

**Results:** The duration of analgesia was significantly prolonged in Group D-X when compared to that in Group X and Group D [(19 h; IQR, 18.5–19.0 h) versus (16 h; IQR, 15.5–16.5 h) versus (13 h, IQR, 12–14 h) *p* value < 0.001]. The duration of sensory block was significantly prolonged in Group D-X compared to that in Group X and Group D [(15 h, IQR, 15‐16 h) versus (13 h, IQR, 12–14 h) versus (10 h, IQR, 10-11 h) *p* value < 0.001]. Similarly, the duration of motor block was prolonged in Group D-X compared to that in Group X and Group D [(16 h; IQR: 16–17.5 h) versus (14 h; IQR; 13–15 h) versus (11 h; IQR: 11-12 h) with significant *p* value < 0.001. Also, the time to onset of sensory and motor block was earlier in Group D-X. The sedation scores were not significant, and no adverse events were observed.

**Conclusion:** Addition of dexamethasone and dexmedetomidine together to a local anesthetic in ultrasound guided costoclavicular block resulted in faster onset with longer analgesic and sensorimotor block duration.

**Trial Registration:** Clinical Trials Registry-India: CTRI/2024/01/061072

## 1. Introduction

Brachial plexus blocks can be performed utilizing various methods such as surface anatomy, paresthesia, electrical neurostimulation, ultrasound guidance, or a combination of these. The attainment of the appropriate level of anesthesia is contingent on the precise deposition and distribution of the local anesthetic within the anatomical structures [1]. The understanding of the relationship between needles, nerves, and the spread of local anesthesia is improved with the use of ultrasonography technology, enabling the exact administration of local anesthetics close to the targeted nerve or nerve plexus [1, 2].

Various techniques can be employed to block the brachial plexus and provide anesthesia to the arm and forearm. These include techniques performed above the clavicle such as the interscalene and supraclavicular block and those performed below the clavicle such as the costoclavicular, infraclavicular, and axillary block. The incidence of hemi-diaphragmatic paresis is higher with more proximal approaches to brachial plexus block compared with the costoclavicular approach, in which the incidence is only about 5% [1]. The costoclavicular approach targets the cords of the brachial plexus at a level below the phrenic nerve, thereby minimizing the risk of inadvertent phrenic nerve blockade [3]. So, there is superior preservation of diaphragmatic contractility and lesser deterioration of pulmonary function tests compared with other proximal approaches to brachial plexus block [3]. The costoclavicular block (CCB) is increasingly used due to the anatomically advantageous configuration of the brachial plexus nerves in the proximal infraclavicular fossa. At this place, the nerves are bundled close together, resulting in a more rapid onset of analgesia compared to the infraclavicular approach.

Dexamethasone is a steroid adjuvant used in peripheral nerve blocks by providing early onset and prolonging the duration of analgesia. It acts as an anti-inflammatory and by inhibiting potassium channel-mediated nociceptive C-fibers [4]. Dexmedetomidine is an alpha 2 adrenergic agonist adjuvant commonly used in peripheral nerve blocks by causing the constriction of the vascular smooth muscle cells to reduce the absorption of local anesthetics. It also has a direct effect on the peripheral nerve by blocking the hyperpolarization-activated cation current [5, 6]. Various studies have assessed the effects of both these adjuvants in interscalene and supraclavicular blocks but we are the first to assess their effects in addition to local anesthetics in the CCB.

The objective of our study was to compare the duration of analgesia, the duration of sensory and motor blocks, and the onset time of sensory and motor blocks by adding adjuvants like dexamethasone and dexmedetomidine to local anesthetics for ultrasound-guided CCB. We hypothesized that combining both dexamethasone with dexmedetomidine will prolong the duration of action and also reduce the onset time of the block when compared to either adjuvant used alone.

## 2. Methods

### 2.1. Trial Design

This was a single-center, double-blinded randomized controlled study conducted from January 2024 to May 2024 conforming to the CONSORT guidelines. Before the start of the study, ethics approval was provided by the Institutional Ethics Committee (CSP—MED/23/MAR/85/69).

### 2.2. Participants

All patients scheduled to undergo below elbow surgeries at Sri Ramachandra Institute of Higher Education and Research were enrolled and assessed for eligibility. Patients between 18 years and 80 years, ASA 1 to 3, and BMI 18–35 kg/m^2^ were included. The patients who did not give consent for the study, who were allergic to drugs used, coagulopathy, sepsis, and neuropathy were excluded from the study. Written informed consent was obtained from all the participants by the primary investigators.

### 2.3. Randomization

Before the start of the study, randomization was done by computer-generated random numbers and sealed enveloped technique. Enrolled patients were allocated to be administered with the local anesthetic mixture of 15 mL of 0.5% bupivacaine and 15 mL of premixed 2% lignocaine with adrenaline 1:200,000 (5 μg/mL) and the allocated adjuvant group mixture. Group D participants received 4 mg dexamethasone. Group X participants received 1 μg/kg dexmedetomidine, and Group D-X received a combination of both 4 mg dexamethasone and 1 μg/kg dexmedetomidine.

### 2.4. Blinding

The participants and investigators were blinded to group allocation.

### 2.5. Intervention

A preanesthetic checkup of patients was done before the surgery, and nil per oral orders were followed. After arriving at the operating room, ASA standard monitors were attached and an intravenous premedication of 2 μg/kg of fentanyl was given. Supplemental oxygen at 6 L/min was given through the Hudson mask.

The block was performed by allowing the patient to lie supine and the arm abducted with the elbow flexed at a 90-degree angle. The head was turned toward the opposite side. A 3–16-MHz, high-frequency, linear ultrasound transducer (model name 9018, SONOSITE, serial no. Q5GQXD Fujifilm) was used to perform the block. The probe was placed horizontally under the clavicle, where the plexus is visualized lateral to the axillary artery. After obtaining the correct alignment, a 21 G, 4-inch (0.80 ∗ 100 mm) Stimuplex, an insulated needle (B. Braun, Melsungen, Germany), was introduced by an in-plane technique from the lateral to the medial side to reach the plexus. Confirmation of the needle is done by observing the motor response to nerve stimulation at 0.5 mA and the allocated local anesthetic adjuvant mixture was injected.

The local anesthetic and allocated adjuvant group mixture were prepared by an anesthesiologist not involved in the study.

  Group D participants received 15 mL of 0.5% bupivacaine, 15 mL of premixed 2% lignocaine with adrenaline 1:200,000 (5 μg/mL), 1 mL of 4 mg dexamethasone, and 1 mL of normal saline (total volume 32 mL).
  Group X participants received 15 mL of 0.5% bupivacaine, 15 mL of premixed 2% lignocaine with adrenaline 1:200,000 (5 μg/mL), and 1 μg/kg dexmedetomidine diluted with 2 mL of normal saline (total volume 32 mL).
  Group D-X participants received 15 mL of 0.5% bupivacaine, 15 mL of premixed 2% lignocaine with adrenaline 1:200,000 (5 μg/mL), 1 mL of 4 mg dexamethasone, and 1 μg/kg dexmedetomidine diluted with 1 mL of normal saline (total volume 32 mL).

The blocks were performed by another anesthesiologist who had an experience of more than 100 ultrasound-guided CCBs and was responsible for the perioperative care of the patients. A different blinded anesthesiologist who was not involved in the study assessed the block parameters and other outcomes while recording data in the data sheet for up to 24 h in the postoperative period.

At the end of the surgery, the patients were shifted to the postanesthesia care unit for observation and moved to their respective wards. Intravenous injection Ketorolac 30 mg was given as the first rescue analgesic when the patient first experienced surgical site pain after documenting the time to request of first rescue analgesic as the analgesic duration. Subsequent postoperative pain management was taken care of by the attending physician as per their discretion and institutional practices.

### 2.6. Data Collection

Baseline demographic data and patient characteristics such as age, sex, weight, BMI, ASA physical status were obtained preoperatively. The musculocutaneous, median, radial, and ulnar nerves were assessed for sensory block using a cold test on a three-point scale [7, 8]. 0 = Absence of block, 1 = Analgesia (able to perceive only touch), 2 = Anesthesia (not able to perceive cold and touch). The lateral side of the forearm (musculocutaneous), palmar aspect of the thumb (median), lateral side of the dorsal aspect of the hand (radial), and palmar aspect of the fifth finger (ulnar) were checked to assess sensory block.

Additionally, a three-point rating system was employed to assess motor block [7, 8] (0 = No block, 1 = Paresis (partial or incomplete paralysis), 2 = Paralysis (complete paralysis). The motor block was assessed by elbow flexion (musculocutaneous), thumb abduction (radial), thumb opposition (median), and thumb adduction (ulnar). The highest possible combined score was 16 points. The block was considered successful if the total score was 14 points, provided that the sensory block achieved a minimum of 7 out of 8 points without the need for intravenous narcotics, general anesthesia or local infiltration or rescue blocks by the surgical team. If the composite score was below 14 after 30 min, the block was considered ineffective, and general anesthesia was administered instead and the patients were excluded from the study.

The onset time was defined as the time required to obtain the total score of 14 points.

In the postoperative period, the duration of sensory block was defined as the first time to regain sensation in the fingers. Also, the duration of motor block was defined as the time to move the fingers on the blocked upper limb. The analgesic duration was defined as the time when the patient first experienced surgical site pain.

A blinded observer also documented any adverse events till the end of surgery such as any arterial puncture, local anesthetic toxicity, respiratory depression, and bradycardia or hypotension. Bradycardia was defined as a heart rate lesser than 50 beats/min, and hypotension was defined as a mean arterial pressure lesser than 60 mm Hg.

Sedation scores were checked by six-point Ramsay sedation scores [9] immediately after surgery, in the PACU, and at 6 h and 12 h postoperatively.

### 2.7. Primary Outcomes

The primary outcome that we studied was the time to administer the first rescue analgesic (analgesic duration).

### 2.8. Secondary Outcomes

The secondary outcomes studied included the duration of sensory and motor blocks, the time to the onset of sensory and motor blocks, intraoperative and postoperative sedation scores, and adverse effects.

### 2.9. Sample Size Calculation

Based on the reported value of the duration of analgesia as per the reference study by Aliste et al. [7], the effect size of 0.366 was derived. With a 5% two-sided alpha error and 90% power of the study, by using the scenario of F-tests and one-way ANOVA of G^∗^power software co-Guide Research Enablement and Productivity (REAP) Version 1.2, the sample size was calculated. Thirty-three study participants were required in each of the three intervention groups. To account for a nonparticipation rate of 5%, another two subjects will be included in each group. Hence, the final sample size is 35 subjects in each of the three intervention groups, with a total sample size of 105.

### 2.10. Statistics Analysis

Categorical variables were represented and analyzed as frequency and proportion. Continuous variables were described as mean ± SD for normally distributed variables and median (IQR) for non-normally distributed variables. The statistical significance between categorical variables such as age, gender, and ASA grade was evaluated using a chi-square test. The quantitative variables that were non-normally distributed such as weight and BMI were expressed as one-way ANOVA to compare the mean ± SD between the three groups. The primary outcome was the time to administer the first rescue analgesia and the duration of the sensory and motor blocks, which was expressed in median (IQR) between the three groups using the Kruskal–Wallis test. Secondary outcomes were time to the onset of block, sedation scores, postoperative pain scores, and adverse effects, which were also expressed in median (IQR) between the three groups using the Kruskal–Wallis test. The sedation scores and postoperative pain scores were analyzed using a chi-square test. The Bonferroni post hoc test was used for pairwise comparison. *P* value < 0.05 was considered statistically significant.

## 3. Results

**Figure 1:**
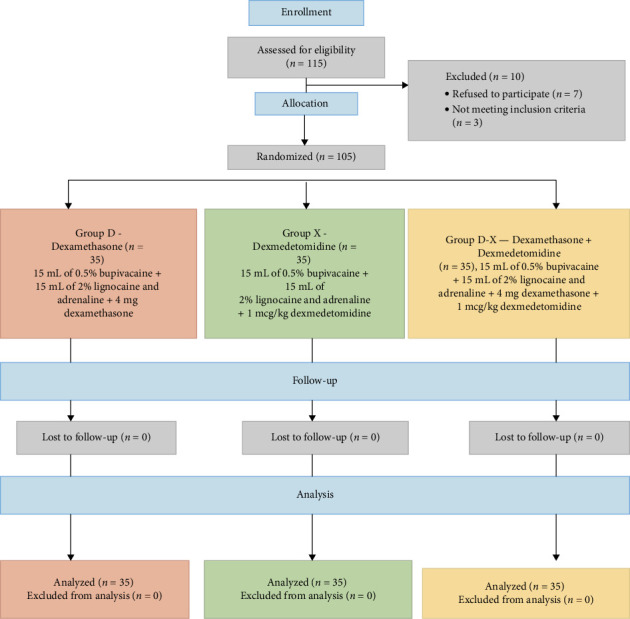
Consolidated standards of reporting trials (CONSORT) chart. *n*: number of patients.

During the study period, 115 patients scheduled for below elbow surgeries were considered. The CONSORT diagram is shown in Figure 1.

After enrollment, 10 patients were excluded and the remaining 105 patients, 35 in each group, were analyzed per protocol. Demographic parameters of age, gender, and BMI distributed across three groups were comparable. The data are shown in Table 1.

  Primary outcomes: The data are shown in Table 2.
  The duration of analgesia in Group D+X was significantly prolonged (19 h; IQR 18.5–19 h) compared to that in Group X (16 h; IQR 15.5–16.5 h) and Group D (13 h; IQR 12–14 h), with significant statistical *p* value < 0.001.
  Secondary outcomes: The data are shown in Table 2.
  The duration of the sensory block was prolonged significantly in Group D+X (15 h; IQR: 15-16 h) compared to that in Group X (13 h; IQR 12–14 h) and Group D (10 h; IQR 10-11 h) with a significant *p* value < 0.001.

Group D had the shortest median duration of motor block (11 h, IQR: 11-12 h), followed by Group X (14 h, IQR: 13–15 h), and Group D+X exhibited the longest duration of motor block (16 h, IQR: 16.0–17.5 h) with a statistically significant *p* value < 0.001.

**Figure 2:**
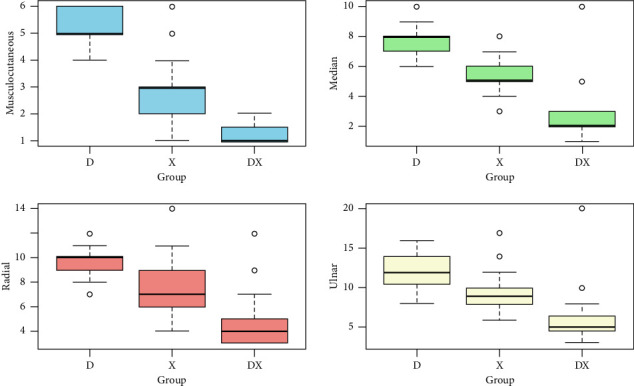
Boxplot of the comparison of the onset of sensory block in minutes for different nerves between the three groups.

The median (IQR) onset time for sensory block of the musculocutaneous was shorter in Group D+X (1 min; IQR 1.0–1.5 min) compared to that in Group X (3 min; IQR 2-3 min) and Group D (5 min; IQR 5-6 min), with a *p* value of < 0.001. Similarly, the onset times for median, radial, and ulnar nerves were also shorter for Group D+X when compared to those in Group X and Group D, and this difference was statistically significant (*p* value < 0.001). The data are shown in Figure 2.

**Figure 3:**
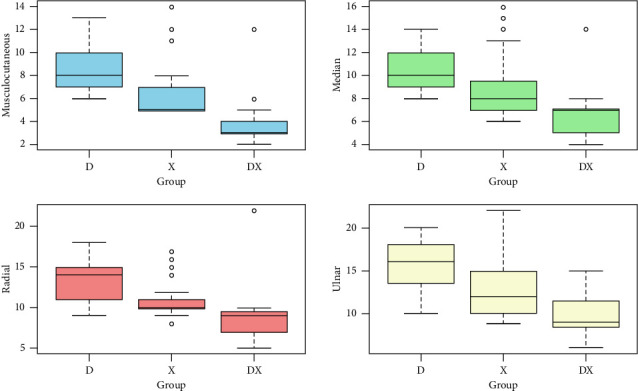
Boxplot of the comparison of the onset of motor block for different nerves in minutes between the three groups.

The onset time of motor block for the musculocutaneous nerve in Group D+X demonstrated the fastest onset (median: 3 min, IQR: 3-4 min), followed by Group X (median: 5 min, IQR: 5–7 min), and Group D, which exhibited the slowest onset (median: 8 min, IQR: 7–10 min), with a *p* value of < 0.001. Similarly, for median, radial, and ulnar nerves, Group D+X consistently displayed the shortest onset times compared to Groups X and D, and the differences were statistically significant (*p* < 0.001). The data are shown in Figure 3.

The sedation scores at different time points varied significantly among groups in our study but no adverse effects were seen. The data are shown in Table 3.

## 4. Discussion

The ultrasound-guided costoclavicular brachial plexus block has captured the interest of anesthesiologists as an improved technique for brachial plexus block in recent years. The area between the central point of the clavicle and the first rib is identified as the costoclavicular space. The three cords of the brachial plexus are compactly grouped lateral to the axillary artery where the plexus is reached immediately caudal to the central point of the clavicle [10]. Because of this compact anatomy, it has a rapid onset of blockade. Cords, which are present in this space, are located more superficially than the cords seen in the traditional approach at the lateral part of the infraclavicular fossa and they are grouped close to each other with a consistent anatomical relationship [11]. It is also considered a retrograde channel in providing anesthesia for suprascapular nerve in shoulder surgeries [12]. The unique features resulting in the CCB are gaining attention as an emerging brachial plexus block technique, with many research studies proving that it can provide a successful and fast onset of the block with a single, small-volume injection of local anesthetic. This approach provides a safe and reliable analgesic effect as it is in a superficial and fixed position with the advantage of real-time ultrasound visualization of the plexus, preventing injury to major vessels and targeted delivery of local anesthetics to the nerves reducing the complications seen with other techniques such as interscalene, supraclavicular, and axillary brachial plexus block.

In this study, we have compared the duration of the action of local anesthetics with dexamethasone (Group D), dexmedetomidine (Group X), and a combination of both dexamethasone and dexmedetomidine (Group D-X) in patients for below elbow surgeries. The results of our study suggested that the combination of both dexamethasone and dexmedetomidine results in a longer duration of analgesia compared to other groups.

Sripriya et al. [13] studied the block onset by comparing the combination of bupivacaine and lignocaine versus bupivacaine alone and concluded that the combined bupivacaine and lignocaine had earlier onset than bupivacaine alone. Therefore, we chose the local anesthetic mixture of bupivacaine and lignocaine plus adrenaline to get an early onset of the block.

Bravo et al. [8] studied the different doses of 2 mg, 5 mg, and 8 mg dexamethasone in the ultrasound guided infraclavicular block and concluded that no differences were observed between the three groups with regard to onset time, sensorimotor, and analgesic durations. To avoid the theoretical risk of neurotoxicity, we chose 4 mg of dexamethasone in our study.

Jung et al. [14] studied 2 μg/kg versus 1 μg/kg of dexmedetomidine for interscalene blocks in arthroscopic surgeries. They found that 2 μg/kg had a longer duration of analgesia than 1 μg/kg, but the incidence of hypotension and bradycardia was found to be higher in the 2 μg/kg group. Hence, we chose a dose of dexmedetomidine 1 μg/kg for our study.

In the Shubha et al. [15] study, the median duration of receiving rescue analgesia was 13 h for the CCB and 12 h for the supraclavicular block. In our study, the longest median duration to receive rescue analgesia was 19 h in Group D+X, compared to Group X, which had a median duration of 16 h. Patients in Group D who received dexamethasone had the shortest median period of obtaining rescue analgesia, which was 13 h. The use of dexamethasone and dexmedetomidine in combination resulted in an extended period of analgesic duration in our study.

Aliste et al. [7] studied the average duration of sensory block in patients who received 35 mL of local anesthetic along with either dexamethasone–dexmedetomidine combination or dexamethasone alone in the infraclavicular block. They found that the dexamethasone–dexmedetomidine combination had a greater duration of sensory block (21 h) as compared to dexamethasone alone (20 h). In our study, Group D+X had an average longer duration of sensory block (15 h) compared to Group D (10 h). Similarly, the duration of the motor block was longer in Group D+X (16 h) compared to that in Group D (11 h). The disparity in the duration of sensory and motor blockades could probably be attributed to the lower volume of local anesthetics used in our study (32 mL).

Iyengar et al. compared dexamethasone with dexmedetomidine as an adjuvant to bupivacaine in the infraclavicular block and found that the group that received a fixed dose of 75 mcg of dexmedetomidine was associated with increased levels of postoperative sedation [16]. In our study, the participants received only 1 mcg/kg of dexmedetomidine (a much lower dose per kilogram) in both Group X and Group D-X. The sedation scores at different time points varied significantly among groups in our study. At 1 h, immediately after surgery, postoperatively at 1 h and postoperatively at 6 h, Group D-X consistently exhibited higher sedation scores compared to Groups D and X. Although there was a trend, the difference in sedation scores among the groups did not reach statistical significance (*p*=0.094) at 12 h postoperatively.

Numerous studies have established that the maximum safe dose of dexmedetomidine is 2 μg/kg, which provides the longest duration of continuous analgesia. However, using dexmedetomidine at doses between 1.5 and 2 μg/kg is associated with a higher incidence of hypotension [17]. Therefore, we selected a low dose of 1 μg/kg of dexmedetomidine, as it offers an optimal balance between effective postoperative analgesia and the potential adverse effects of peripheral nerve block. Additionally, the use of adrenaline in a local anesthetic mixture for brachial plexus blockade can increase the risk of hypotension, especially when used alongside an intravenous infusion of dexmedetomidine [18]. However, in our study, we did not observe this issue, as we administered dexmedetomidine as a perineural adjunct. This combination of local anesthetic mixture with adrenaline and dexmedetomidine has already been safely used in numerous studies [19, 20].

Hong B et al. [21] studied the occurrence of hemi-diaphragmatic paralysis between costoclavicular and supraclavicular brachial plexus blocks. The occurrence of hemi-diaphragmatic paralysis was lower (2.5%) in individuals who received CCB compared to patients who received supraclavicular block (39.8%). They discovered that the drug's volume also plays a role in causing hemi-diaphragmatic paralysis. Our study did not evaluate sonographic diaphragmatic excursions or assess diaphragmatic thickness fraction, which is a constraint of our research.

### 4.1. Limitations

The sensory duration, motor duration, and analgesic duration were documented based on the recall by the patients. Rescue analgesic was administered once the patients reported pain rather than time-based postoperative pain scores as the uniformity of pain threshold across groups could not be ensured. Also, the postoperative follow-up was restricted to 24 h only.

## 5. Conclusion

The combination of dexamethasone and dexmedetomidine as adjuvants along with local anesthetic provides a longer duration of analgesia with a faster onset of sensorimotor block when compared to individual adjuvants in ultrasound-guided single-shot CCBs for below elbow surgeries. Further studies with larger sample sizes are required to identify the optimal dosing combination of dexamethasone and dexmedetomidine.

## Figures and Tables

**Table 1:** Baseline demographic characteristics for study participants who received local anesthetic mixture with adjuvants dexamethasone, dexmedetomidine, and a combination of dexamethasone and dexmedetomidine.

Variable	Group D (*n* = 35)	Group X (*n* = 35)	Group D-X (*n* = 35)	*p*–value
Age (years)	44.31 ± 13.93	48.94 ± 14.7	45.29 ± 12.36	0.333

*Gender*				
Male	13 (37.1%)	19 (54.3%)	19 (54.3%)	0.253
Female	22 (62.9%)	16 (45.7%)	16 (45.7%)	

BMI (kg/m^2^)	26.94 ± 3.1	26.2 ± 3.11	25.97 ± 3.43	0.421

*ASA grade*				
ASA I	13 (37.1%)	23 (65.7%)	13 (37.1%)	0.022^∗∗^
ASA II	22 (62.9%)	12 (34.3%)	22 (62.9%)	
ASA III	0	0	0	

*Note: n* = number. Group D = bupivacaine + dexamethasone, Group X = bupivacaine + dexmedetomidine, Group D-X = bupivacaine + dexamethasone and dexmedetomidine. Data values for age and BMI are expressed as mean ± SD; data values for gender and ASA grade are expressed as number (%) of patients.

Abbreviations: ASA, American Society of Anaesthesiologists; BMI, body mass index; SD, standard deviation.

^∗∗^ *p* value less than 0.05 was considered significant.

**Table 2:** Comparison of costoclavicular brachial plexus block characteristics between the groups.

Parameter	Group D (*n* = 35)	Group X (*n* = 35)	Group D-X (*n* = 35)	*p*–value
Analgesic duration (h)	13 (12, 14)	16 (15.5, 16.5)	19 (18.5, 19.0)	< 0.001^∗∗^
Duration of sensory block (h)	10 (10, 11)	13 (12, 14)	15 (15, 16)	< 0.001^∗∗^
Duration of motor block (h)	11 (11, 12)	14 (13, 15)	16 (16.0, 17.5)	< 0.001^∗∗^

*Onset of sensory block (min)*				
Musculocutaneous	5.0 (5.0, 6.0)	3.0 (2.0, 3.0)	1.0 (1.0, 1.5)	< 0.001^∗∗^
Median	8.0 (7.0, 8.0)	5.0 (5.0, 6.0)	2.0 (2.0, 3.0)	< 0.001^∗∗^
Radial	10 (9.0, 10)	7.0 (6.0, 9.0)	4.0 (3.0, 5.0)	< 0.001^∗∗^
Ulnar	12 (10.5, 14.0)	9.0 (8.0, 10)	5.0 (4.5, 6.5)	< 0.001^∗∗^

*Onset of motor block (min)*				
Musculocutaneous	8.0 (7.0, 10)	5.0 (5.0, 7.0)	3.0 (3.0, 4.0)	< 0.001^∗∗^
Median	10 (9.0, 12)	8.0 (7.0, 9.5)	7.0 (5.0, 7.0)	< 0.001^∗∗^
Radial	14 (11, 15)	10 (10, 11)	9.0 (7.0, 9.5)	< 0.001^∗∗^
Ulnar	16 (13.5, 18.0)	12 (10, 15)	9.0 (8.5, 11.5)	< 0.001^∗∗^

*Note:* Group D = bupivacaine + dexamethasone, Group X = bupivacaine + dexmedetomidine, Group D-X = bupivacaine + dexamethasone and dexmedetomidine. Data are presented as median (interquartile range), *n* = number.

^∗∗^Value of *p* less than 0.01 was considered highly significant.

**Table 3:** Comparison of sedation scores between the groups.

Parameter	Group D (*n* = 35)	Group X (*n* = 35)	Group D-X (*n* = 35)	*p*–value
At 1 h intraoperatively	1 (1, 2)	2 (2, 2)	3 (2, 3)	< 0.001^∗∗^
Immediately after surgery	1 (1, 2)	2 (1.5, 2.0)	2 (1.5, 2.0)	< 0.001^∗∗^
Postoperative at 1 h	1 (1, 2)	1 (1, 2)	2 (1, 2)	0.001^∗∗^
Postoperative at 6 h	1 (1, 2)	1 (1, 2)	2 (1.5, 2.0)	0.001^∗∗^
Postoperative at 12 h	1 (1, 1)	1 (1, 2)	1 (1, 2)	0.094

*Note:* Group D = bupivacaine + dexamethasone, Group X = bupivacaine + dexmedetomidine, Group D-X = bupivacaine + dexamethasone and dexmedetomidine. Data are presented as median (interquartile range), *n* = number.

^∗∗^Value of *p* less than 0.01 was considered highly significant.

## Data Availability

Research data are not shared due to privacy or ethical considerations.
